# Use of Mendelian Randomization to Examine Causal Inference in Osteoporosis

**DOI:** 10.3389/fendo.2019.00807

**Published:** 2019-11-21

**Authors:** Jie Zheng, Monika Frysz, John P. Kemp, David M. Evans, George Davey Smith, Jonathan H. Tobias

**Affiliations:** ^1^MRC Integrative Epidemiology Unit (IEU), Population Health Sciences, Bristol Medical School, University of Bristol, Bristol, United Kingdom; ^2^Musculoskeletal Research Unit, Translational Health Sciences, Bristol Medical School, University of Bristol, Bristol, United Kingdom; ^3^The University of Queensland Diamantina Institute, The University of Queensland, Woolloongabba, QLD, Australia

**Keywords:** bone mineral density (BMD), fractures - bone, pleiotropy, sclerostin, GWAS - genome-wide association study

## Abstract

Epidemiological studies have identified many risk factors for osteoporosis, however it is unclear whether these observational associations reflect true causal effects, or the effects of latent confounding or reverse causality. Mendelian randomization (MR) enables causal relationships to be evaluated, by examining the relationship between genetic susceptibility to the risk factor in question, and the disease outcome of interest. This has been facilitated by the development of two-sample MR analysis, where the exposure and outcome are measured in different studies, and by exploiting summary result statistics from large well-powered genome-wide association studies that are available for thousands of traits. Though MR has several inherent limitations, the field is rapidly evolving and at least 14 methodological extensions have been developed to overcome these. The present paper aims to discuss some of the limitations in the MR analytical framework, and how this method has been applied to the osteoporosis field, helping to reinforce conclusions about causality, and discovering potential new regulatory pathways, exemplified by our recent MR study of sclerostin.

## Introduction

Osteoporosis is a common disorder leading to skeletal fragility and increased fracture risk. This condition is strongly influenced by age and sex, as well as genetic factors. Establishing which risk factors play a causal role in osteoporosis is helpful in unraveling pathogenic mechanisms, and in identifying potential new preventative and treatment strategies. Epidemiology studies in the osteoporosis field have examined relationships between putative risk factors and fracture risk, the main clinical consequence of osteoporosis. Investigations have also studied risk factors for bone mineral density (BMD) as measured by DXA, which is a strong predictor of fracture risk (1). Traditional observational studies have reported that a range of potentially modifiable risk factors, including sex-steroid deficiency, low body mass index (BMI), physical inactivity, smoking, heavy alcohol consumption, and low calcium and vitamin D, are related to BMD and fractures. However, studies of this type suffer from confounding and reverse causality (2, 3). Randomized controlled trials (RCTs) are the gold standard for inferring causality, because they are unaffected by these issues if performed correctly. However, RCTs are expensive, resource-intensive, time consuming, and may have important ethical limitations.

**Figure 1:**
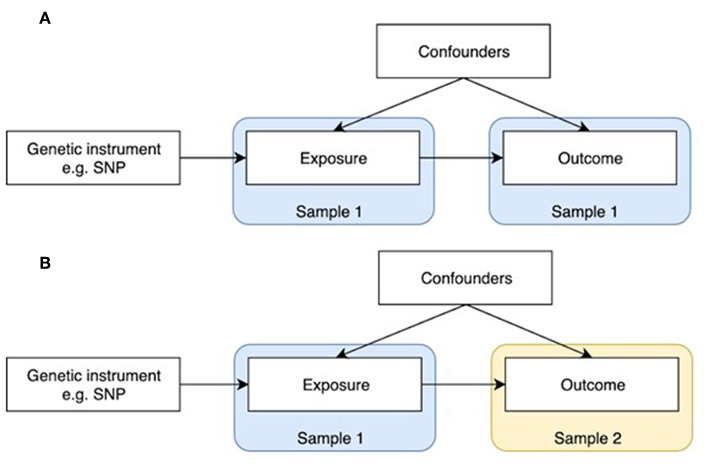
One-sample and two-sample Mendelian randomization study designs. **(A)** One-sample Mendelian randomization is based on a population where both exposure and outcome have been measured. **(B)** In two-sample Mendelian randomization, exposures and outcomes are measured in non-overlapping populations. SNP-exposure is derived in Sample 1, and SNP-outcome in Sample 2.

MR is a statistical method for inferring causality which is analogous to an RCT, except that genotypes are used to randomize participants into different levels of the exposure/treatment. MR can be implemented as a form of instrumental variables analysis, where genetic variants, normally single nucleotide polymorphisms (SNPs), are used as proxies (“instruments”) for the exposure of interest (see Figure 1) (4, 5). According to Mendel's Laws of Inheritance, alleles segregate randomly when passed from parents to offspring. According to his (second) Law of Independent Assortment, which forms the foundation of MR, the inheritance of one pair of factors (genes) is independent of the inheritance of the other pair. Thus, offspring genotypes are unlikely to be associated with confounders in the population. In addition, since germ-line genotypes are determined at conception, they precede outcomes being investigated, and so observed associations cannot be explained by reverse causation. However, unlike RCTs which generally involve relatively short term interventions, genetic influences exert their effects from conception onwards, and so causal effects estimated from MR represent life-long exposures.

MR was initially developed in the form of one-sample MR, which relies on access to individual level data (Figure 1A). One limitation of this method is that most individual cohorts do not have many traits measured simultaneously. Two sample MR was subsequently developed to overcome this issue by using summary level data derived from independent cohorts that collectively have many exposures and outcomes measured (Figure 1B). GWAS data is now available on thousands of plausible osteoporosis risk factors which can be leveraged by two sample MR. The extensive opportunities to explore causal influences on bone phenotypes using the MR approach is summarized in two recent reviews (2, 5).

Though previous MR studies have contributed to our understanding of causal factors involved in the etiology of osteoporosis, as discussed below, MR has a number of inherent assumptions and limitations, for which a range of sensitivity analyses have been developed (6–8). MR analyses may also be subject to several sources of bias (9). For example, if individuals with a certain disease outcome are drawn from a population with distinct ancestry to disease-free controls, this may lead to differences in frequency of genetic variants between those with and without disease, and hence spurious associations with genotypes related to putative risk factors. Furthermore, dynastic effects need to be considered for many traits, including BMD, whereby effects of genetic variation in the offspring are partly mediated by shared parental genetic influences acting via early life environment (10). The present paper aims to discuss how MR has been applied to the osteoporosis field, including the approaches taken to address limitations in the MR analytical framework.

## Osteoporosis Outcomes

Consistent with epidemiology studies of osteoporosis in general, the majority of MR studies in osteoporosis have utilized DXA-measured BMD as the outcome, which is widely used clinically as the gold standard for diagnosing osteoporosis. As well as being predictive of the clinical consequence of osteoporosis, namely fractures (1), BMD has a major heritable component, making it a highly suitable outcome for MR analyses (11, 12). Initial studies utilized summary statistics from GWASs, such as those based on the GEnetic Factors for OSteoporosis Consortium (GEFOS, http://www.gefos.org/), which made GWAS findings for DXA-measured BMD publicly available for a range of sites including the lumbar spine (LS) and femoral neck (FN) (13). Whereas, GEFOS has the advantage of providing BMD GWAS data for multiple skeletal sites, a potential disadvantage is that the latter GWAS adjusted for weight. The justification for this is that areal BMD measured by DXA is influenced by body size, which is partly accounted for by adjusting for weight. However, this can have unintended consequences, such as the introduction of spurious genetic associations as a consequence of collider bias (14). In addition, by using BMD summary statistics corrected for weight, subsequent MR analysis may be biased as effects on BMD mediated by weight may not be accurately estimated. Moreover, as described below, use of GWAS outputs that were adjusted for weight and/or height may complicate interpretation when applying these data in MR studies examining relationships between BMI and BMD. The latter relationship is thought to be relatively complex, involving both a mass effect leading to greater loading acting via the mechanosensor, and shared endocrine pathways.

Recently published GWASs for estimated BMD (eBMD) derived from heel ultrasound in UK Biobank (11, 12) have the advantage that both unadjusted and adjusted summary statistics are available on request, enabling sensitivity analyses to be performed. Moreover, the large sample size provides a significant advance in terms of power, which is one of the major limitations of MR studies. Ultrasound derived BMD does not involve radiation, is quick and cheap, and is therefore well-suited to population studies involving hundreds of thousands of people. The limitation is that estimated BMD is not well-understood and we are not entirely sure how well it proxies BMD. That said, eBMD and DXA-BMD measures are reasonably highly correlated genetically (*r* = ~0.5), as are eBMD and fractures (*r* = ~0.5) (11), and ultrasound-derived measures have previously been reported to predict subsequent fractures with similar accuracy to DXA BMD (15–18).

BMD is an intermediary phenotype; low BMD is only of pathological significance as a result of its causal relationship with fracture. However, since many risk factors for osteoporosis act via BMD, their relationship with BMD is somewhat stronger than that with fracture, with the result that MR studies using fracture as the outcome tend to be underpowered. That said, in a large MR study of fractures based on discovery set of 37,857 fracture cases and 227,116 controls, with replication in 147,200 fracture cases and 150,085 controls, Trajanoska et al. found that higher BMD had an expected causal effect in reducing fractures (19). Moreover, Morris et al. identified 13 bone fracture loci in approximately 1.2 million individuals, all of which were associated with eBMD (12). It may also be possible to extend MR studies in osteoporosis to examine causal effects on other phenotypes relevant to osteoporosis. BMD is not the sole causal determinant of fracture, and GWAS signals have recently been identified for several geometric parameters derived from hip DXA, which are also thought to be related to fracture risk (20). GWAS efforts are also underway for osteocalcin and CTX, offering opportunities for MR studies to examine causal pathways for other outcomes contributing to fracture risk, such as bone turnover.

## Osteoporosis Risk Factors

A range of risk factors for osteoporosis identified in epidemiological studies have been examined in MR studies intended to explore their causal effects, using BMD as the outcome, the majority of which have yielded no or weak evidence of causality (see Table 1). For example, studies using BMD as an outcome did not find support for a causal effect of vitamin D (21–23) or genetically determined calcium intake as reflected by lactase persistence genotype (24). Guo et al. found no evidence to suggest a causal effect of alcohol consumption on BMD whereas smoking status was found to be causally related to lower BMD (25); however, it should be noted that smoking (exposure) and eBMD (outcome) instruments were derived from the same sample population which could result in biased estimates (8). In a subsequent study, genetic predisposition to smoking initiation was associated with fracture risk, but not eBMD; genetic liability to alcohol dependence was also associated with fracture and lower eBMD, whereas no association was seen for genetically predicted alcohol intake (26). However, this study also included UK Biobank participants in exposure and outcome instruments which could lead to bias. Other studies exploring the effect of serum urate (27, 28), inflammatory markers (29) and thyroid stimulating hormone (30) found no evidence for association with FN or LS BMD. Rather than a risk factor, MR analysis suggests that lowering LDL-C levels and statin therapy improve BMD (31).

**Table 1:** Examples of studies investigating causal associations between risk factors and BMD.

**Exposure**	**Sample source for exposure**	**Genetic variants (*n*)**	**Outcome**	**Sample size and data source for outcome**	**Method**	**Evidence of causal effect (Yes/No)**	**References**
Vitamin D	Chinese populations	10	LS BMD	Postmenopausal Chinese women, *N* = 1,824	One-sample	No	(21)
		10	FN BMD				
		10	Total hip BMD				
		10	LS BMD				
		10	FN BMD				
		10	Total hip BMD				
Vitamin D	Europeans, *N =* 79,366	6	TB BMD	Individuals from Europe (86%), America (2%) and Australia (14%), *N =* 66,628	Two-sample (IVW)	No	(22)
Vitamin D	Europeans, *N =* 42,274 (SUNLIGHT consortium)	5	DXA FN BMD	Europeans, *N =* 32,965 (GEFOS Consortium)	Two-sample (weighted median)	No	(23)
		5	DXA LS BMD				
		5	eBMD	Europeans, *N =* 142,487 (UK Biobank)			
Milk intake	Lactase persistence SNP in the *MCM6* gene, based on previous studies	1	Forearm BMD	Europeans, *N =* 53,236 (GEFOS Consortium)	Two-sample (Wald estimate)	No	(24)
		1	FN BMD				
		1	LS BMD				
Alcohol consumption	Europeans	6	FN BMD	Europeans, *N =* 32,735 (GEFOS Consortium)	Two-sample (IVW)	No	(25)
		6	LS BMD	Europeans, *N =* 28,498 (GEFOS Consortium)			
		6	Forearm BMD	Europeans, *N =* 8,143 (GEFOS Consortium)			
		5	Heel BMD	Europeans, *N =* 445,921 (UK Biobank)			
Smoking status	Europeans (including UKBB results)	142	FN BMD	Europeans, *N =* 32,735 (GEFOS Consortium)	Two-sample (IVW)	No	
		142	LS BMD	Europeans, *N =* 28,498 (GEFOS Consortium)			
		139	Forearm BMD	Europeans, *N =* 8,143 (GEFOS Consortium)			
		142	Heel BMD	Europeans, *N =* 445,921 (UK Biobank)	Two-sample (IVW)	Some evidence but could be biased	
Smoking initiation	Europeans (including UKBB results)	1	FN BMD	Europeans, *N =* 32,735 (GEFOS Consortium)	Two-sample (IVW)	No	
		1	LS BMD	Europeans, *N =* 28,498 (GEFOS Consortium)			
		1	Forearm BMD	Europeans, *N =* 8,143 (GEFOS Consortium)			
		1	Heel BMD	Europeans, *N =* 445,921 (UK Biobank)	Two-sample (IVW)	Some evidence but could be biased	
No. of cigarettes smoked per day (CPD)	Europeans (Tobacco and Genetics Consortium)	3	FN BMD	Europeans, *N =* 32,735 (GEFOS Consortium)	Two-sample (IVW)	Very weak evidence	
		3	LS BMD	Europeans, *N =* 28,498 (GEFOS Consortium)	Two-sample (IVW)	No	
		3	Forearm BMD	Europeans, *N =* 8,143 (GEFOS Consortium)		No	
		3	Heel BMD	Europeans, *N =* 445,921 (UK Biobank)		No	
Smoking initiation	Europeans *N =* 1,232,091 (including UK Biobank)	376	eBMD	Europeans, *N =* 426,824 (UK Biobank)	Two-sample (IVW)	No	(26)
			DXA derived BMD	Europeans *N =* 32,965 (GEFOS Consortium)	Two-sample (IVW)	No	
Genetically predicted alcohol intake	Europeans *N =* 941,280 (including UK Biobank)	96	eBMD	Europeans, *N =* 426,824 (UK Biobank)	Two-sample (IVW)	No	
			DXA derived BMD	Europeans *N =* 32,965 (GEFOS Consortium)	Two-sample (IVW)	No	
Genetic liability to alcohol dependence	Europeans *N =* 46,568 (11,569 cases and 34,999 controls)	1	eBMD	Europeans, *N =* 426,824 (UK Biobank)	Two-sample (IVW)	Yes	
		1	DXA derived BMD	Europeans *N =* 32,965 (GEFOS Consortium)	Two-sample (IVW)	No	
Serum urate	Europeans	5	LS BMD	1,322 postmenopausal women and elderly men from Shanghai	One-sample	No	(27)
		5	FN BMD				
		5	Total hip BMD				
Serum urate	Europeans	3	Total hip BMD	Generation 3 cohort in the Framingham Heart Study (*N =* 2,501)	One-sample	No	(28)
		3	FN BMD				
		3	LS BMD				
Inflammatory markers - hsCRP	Europeans	16	Forearm BMD	Europeans, *N =* 32,965 (GEFOS Consortium)	Two-sample (IVW)	No	(29)
		16	FN BMD				
		16	LS BMD				
Thyroid Stimulating Hormone	Europeans, *N =* 26,420	20	FN BMD	Europeans, *N =* 28,498 (GEFOS Consortium)	Two-sample (IVW)	No	(30)
		20	LS BMD				
low LDL-C levels	Global Lipids Genetics Consortium *N =* 188,577	76	TB BMD	Populations from America, Europe and Australia *N =* 66,628	Two-sample (IVW)	Some evidence	(31)
					Multivariable IVW	No	
		76	eBMD	Europeans, *N =* 142,487 (UK Biobank)	Two-sample (IVW)	Yes	
					Multivariable IVW	Yes	
Gene encoding molecular target of LDL-C-lowering therapy (HMGCR)	Global Lipids Genetics Consortium *N =* 188,577	3	TB BMD	Populations from America, Europe and Australia *N =* 66,628	Two-sample (IVW)	Yes	
		3	eBMD	Europeans, *N =* 142,487 (UK Biobank)	Two-sample (IVW)	Yes	
AAM	European women ReproGen Consortium *N =* 182,416	116	LS BMD	GEFOS Consortium (*N =* 53,236) (both males and females)	Two-sample (IVW)	Yes	(32)
		116	FN BMD				
AAM on aBMD in adolescent girls	ReproGen Consortium	331	LS BMD	aBMD in childhood/ adolescence (BMDCS)	Two-sample (FE meta-analysis)	Yes	(33)
		331	FN BMD			No	
		331	Distal radius			No	
AAM on aBMD in adult women	ReproGen Consortium	309	LS BMD	GEFOS Consortium	Two-sample (FE meta-analysis)	Yes	
		309	FN BMD			Yes	
		309	Distal radius			No	
AVB on aBMD in adolescent boys	ReproGen Consortium	43	LS BMD	aBMD in childhood/ adolescence (BMDCS)	Two-sample (FE meta-analysis)	No	
		43	FN BMD			No	
		43	Distal radius			No	
AVB on aBMD in adult men	ReproGen Consortium	42	LS BMD	GEFOS Consortium	Two-sample (FE meta-analysis)	Yes	
		42	FN BMD			Yes	
		42	Distal radius			No	
BMI	Europeans	32	SK-BMD	Europeans, *N =* 5,221 (ALSPAC cohort) *N = 4,223 for SK-BMD	One-sample	No	(34)
		32	UL-BMD			Yes	
		32	LL-BMD			Yes	
		32	SP-BMD			Yes	
		32	PE-BMD			Yes	
Fat mass	Europeans	32	SK-BMD	Europeans, *N =* 5,221 (ALSPAC cohort) *N = 4,223 for SK-BMD	One-sample	No	
		32	UL-BMD			Yes	
		32	LL-BMD			Yes	
		32	SP-BMD			Yes	
		32	PE-BMD			Yes	
Fat mass	Europeans	32	SK-BMD	Europeans, *N =* 5,221 (ALSPAC cohort) *N = 4,223 for SK-BMD	One-sample multivariable MR	No	
		32	UL-BMD			No	
		32	LL-BMD			Yes	
		32	SP-BMD			Yes	
		32	PE-BMD			Yes	
Lean mass	Europeans	32	SK-BMD	Europeans, *N =* 5,221 (ALSPAC cohort) *N = 4,223 for SK-BMD	One-sample multivariable MR	No	
		32	UL-BMD			Yes	
		32	LL-BMD			Yes	
		32	SP-BMD			No	
		32	PE-BMD			Yes	
BMI	GIANT consortium	77	FN BMD	Europeans, GEFOS 2012	Two-sample (IVW)	No	(35)
		77	LS BMD			No	
BMI	East Asian populations	13	Weight-bearing bones	Men, *N =* 1,110	One-sample	Yes	(36)
		13	Non–weight-bearing bones			Yes	
		13	Skull			No	
		13	Weight-bearing bones	Premenopausal women, *N =* 1,015	One-sample	Yes	
		13	Non–weight-bearing bones			No	
		13	Skull			No	
		13	Weight-bearing bones	Postmenopausal women, *N =* 32	One-sample	No	
		13	Non–weight-bearing bones			No	
		13	Skull			No	
T2D	DIAGRAM: 26,676 T2D cases and 132,532 controls	94	eBMD	~150,000 UK Biobank participants	Two-sample (IVW)	No	(37)
CHD	CARDIoGRAMplusC4D	52	eBMD	~150,000 UK Biobank participants	Two-sample (IVW)	No	
T2D	DIAGRAM consortium	32	FN BMD	GEFOS, *N =* 83,894	Two-sample (IVW)	Weak evidence	(35)
		32	LS BMD			No	
Metabolites	Europeans	481 blood metabolites	Hip BMD	2,286 unrelated white subjects for the discovery samples	Pearson correlation	Associations between BMD and 54 blood metabolites	(38)
Total serum calcium	Europeans (discovery cohort *N =* 39,400, replication cohort *N =* 21,676)	7	eBMD	Europeans, *N =* 426,824 (UK Biobank)	Two-sample (IVW)	No	(39)

*LS, lumbar spine; FN, femoral neck; TB, total body; eBMD, estimated bone mineral density; AAM, age at menarche; AVB, age at voice break; UL, upper limbs; LL, lower limbs; SP, spine; PE, pelvis; IVW, inverse-variance weighted; T2D, type 2 diabetes; CHD, coronary heart disease; BMI, body mass index; FE, fixed-effects*.

In terms of constitutive factors, a causal association was observed between later age at menarche and reduced FN and LS BMD in adults (32), and reduced LS BMD in adolescents (33). A study in children found a causal association between BMI/adiposity and BMD (34). A previous study in adults using summary data from GWASs in Europeans found no evidence of a causal effect of BMI (based on 77 SNPs) on FN or LS BMD, however since FN and LS BMD were corrected for weight prior to the GWAS, the variation in BMD attributable to BMI may not be adequately captured by MR analysis (35). In contrast, a one-sample MR in Koreans using 13 BMI-associated SNPs identified in a GWAS of east Asians was suggestive of a causal effect of BMI on BMD on weight bearing sites in men and pre-menopausal women (36). Observation studies have implicated several diseases in the development of osteoporosis, however MR has subsequently found no causal effect of type 2 diabetes (T2D) and coronary heart disease (CHD) on eBMD (37). Another study reported a weak association between increased T2D risk and increased FN BMD, whereas no association was seen with LS BMD (35). In terms of other constitutional factors, genetic predisposition to increased calcium levels was recently found to be unrelated either to eBMD or fracture risk in UK Biobank (39).

Several MR studies have examined osteoporosis risk factors with fracture as the outcome (Table 2). In the study based on UK Biobank from Trajanoska et al. (19), while confirming the expected protective effect of higher BMD on fractures, there was little evidence to suggest a causal effect of dietary factors (vitamin D levels and calcium intake), early menopause, late puberty and range of diseases (including type 1 and 2 diabetes, CHD and inflammatory bowel disease) on risk of fracture. These findings are consistent with results from the above MR studies based on BMD. However, the study did provide some evidence for causal effect of decreased grip strength on fracture risk. A study in 97,811 Danish individuals failed to provide evidence for a relationship between calcium intake and hip fracture (40). Interestingly, an MR study investigating the causal effect of height with 50 diseases reported that one SD increase in genetically determined height was associated with increased risk of hip fracture (41). In terms of the effect of serum hormones, a previous MR study in men reported lower levels of estradiol to be causally related to increased risk of fracture (including all self-reported fractures, major non-vertebral osteoporotic fractures and wrist fractures), whilst there was no evidence for causal association between serum testosterone and fracture risk (42). Furthermore, using a genetic risk score for CRP levels in a Rotterdam study, there was no evidence to support a causal effect of CRP on fracture risk (43).

**Table 2:** Examples of MR studies using fracture as an outcome.

**Exposure**	**Sample source for exposure data**	**Genetic variants (*n*)**	**Outcome**	**Sample size and data sources for the outcome data**	**MR method**	**Evidence of causal effect (Yes/No)**	**References**
Decreased FN BMD	Europeans	43	Fractures at any skeletal site confirmed by medical, radiological, or questionnaire reports	147,200 cases and 150,085 controls (primarily of European ancestry)	Two-sample (IVW)	Yes	(19)
Decreased LS BMD		40				Yes	
Earlier menopause		54				No	
Rheumatoid arthritis		30				No	
Inflammatory bowel disease		19				No	
Type 1 diabetes		151				No	
Decreased THS		20				No	
Homocysteine		13				No	
Decreased Grip strength		15				Yes	
Late puberty		106				Some evidence	
Fasting glucose		35				No	
Coronary heart disease		38				No	
Type 2 diabetes		38				No	
Vitamin D		4				No	
Dairy calcium intake		1				No	
Lactase persistence LCT-13910 C/T genetic variant	Northern Europeans	1	Hip fracture	97,811 Danish individuals	Fixed effects meta-analysis	No	(39)
Height	Europeans, *N =* 253,288 (GIANT)	697	Hip fracture	2,451 fracture cases of 417,434 individuals from UK Biobank	Two-sample (IVW)	Yes	(40)
Serum estradiol	Europeans Europeans	2	All self-reported fractures	Europeans, *N =* 17,650 (UK Biobank)	Two-sample (IVW)	Yes	(41)
		2	Major nonvertebral osteoporotic fractures	(*N =* 4,379; wrist, arm, and hip)			
		2	Wrist fractures	(*N =* 2,637)			
Testosterone		3	All self-reported fractures	(*N =* 17,650)		No	
		3	Major nonvertebral osteoporotic fractures	(*N =* 4,379; wrist, arm, and hip)			
		3	Wrist fractures	(*N =* 2,637)			
Serum CRP levels	Europeans	29	Any fracture	6,386 participants (59% women), of whom 1,561 sustained a fracture	One-sample	No	(42)
Smoking initiation	Europeans *N =* 1,232,091 (including UK Biobank)	377	Any fracture (excluding skull, face, hands and feet, pathological fractures due to malignancy, atypical femoral fractures, periprosthetic, and healed fracture) and any self-reported fractures	Europeans *N =* 426,795 (53,184 cases and 373,611 non-cases) (UK Biobank)	Two-sample (IVW)	Yes	(26)
Genetically predicted alcohol intake	Europeans *N =* 941,280 (including UK Biobank)	99	Any fracture (excluding skull, face, hands and feet, pathological fractures due to malignancy, atypical femoral fractures, periprosthetic, and healed fracture) and any self-reported fractures	Europeans *N =* 426,795 (53,184 cases and 373,611 non-cases) (UK Biobank)	Two-sample (IVW)	No	
Genetic liability to alcohol dependence	Europeans *N =* 46,568 (11,569 cases and 34,999 controls)	2	Any fracture (excluding skull, face, hands and feet, pathological fractures due to malignancy, atypical femoral fractures, periprosthetic, and healed fracture) and any self-reported fractures	Europeans *N =* 426,795 (53,184 cases and 373,611 non-cases) (UK Biobank)	Two-sample (IVW)	Some evidence	
LDL-C levels	*N =* 188,577 (GLSC)	76	Fractures at any skeletal site confirmed by medical, radiological, or questionnaire reports	147,200 cases and 150,085 controls (primarily of European ancestry)	Two-sample (IVW)	No	(32)
Gene encoding molecular target of LDL-C-lowering therapy (HMGCR)	*N =* 188,577 (GLSC)	76	Fractures at any skeletal site confirmed by medical, radiological, or questionnaire reports	147,200 cases and 150,085 controls (primarily of European ancestry)		No	
Total serum calcium	Europeans (discovery cohort *N =* 39,400, replication cohort *N =* 21,676)	6	Fracture	76,549 cases and 470,164 controls from GEFOS, EPIC-Norfolk study and UK Biobank	Two-sample (IVW)	No	(38)

*OR, Odds ratio; IVW, inverse-variance weighted; HR, hazard ratio; THS, thyroid stimulating hormone; LS, lumbar spine; FN, femoral neck*.

Some MR studies have set out to test hypothesized causal effects of BMD on other outcomes. For example, in a study which used summary statistics from the first release of the UK Biobank data (*N* = 11,650), the authors reported some evidence for a causal effect of eBMD on T2D, CHD, HDL-c, and HOMA-IR, testing reciprocal associations for two traits (T2D and CHD), for which there was no evidence of a causal effect on BMD (37). The most recent study in 426,824 UK Biobank participants identified 518 loci associated with eBMD, explaining 20% of its variance (12), meaning that many powerful and robust instruments for MR analyses examining causal effects of eBMD will be available.

## Addressing Pleiotropy

Key points:-

1. Vertical pleiotropy, when the genetic variant has an effect on two or more traits that both influence the outcome via the same biological pathway, is usually not problematic for MR analyses
2. In contrast, horizontal pleiotropy, when a genetic variant is associated with two traits which influence the outcome via independent biological pathways, violates one of the key MR assumptions
3. Several methods have been developed that relax the strict requirement that genetic instruments exhibit no horizontal pleiotropy yet still produce consistent causal effect estimates
4. Where genetic instruments are known to be pleiotropically associated with multiple correlated phenotypes, it may be possible to examine independent effects through exclusion of certain SNPs, or use of multivariable MR.

**Figure 2:**
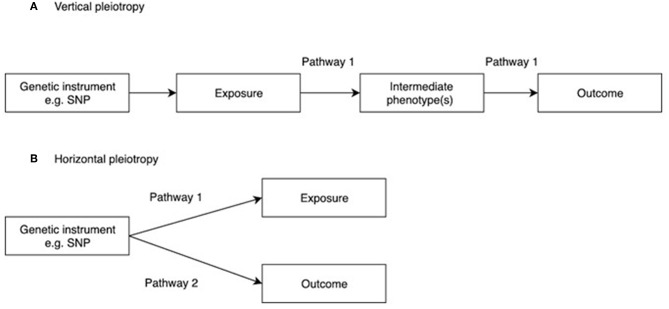
Vertical and horizontal pleiotropy. **(A)** Vertical pleiotropy, which does not violate the MR assumption; **(B)** Horizontal pleiotropy, which violates the MR assumption.

One of the main assumptions of MR is that genetic instruments are only associated with the interest via the exposure being tested. This is known as the “no pleiotropy” assumption or the “exclusion restriction criterion.” When performing an MR study, it is usually unclear whether such an assumption holds. Therefore, various sensitivity analyses are applied to detect the existence of pleiotropy, and to estimate the un-biased causal effect of the exposure on the outcome. Vertical pleiotropy (i.e., a genetic variant has an effect on two or more traits that both influence the outcome via the same biological pathway) is not generally an issue for MR analysis (Figure 2A) (8). However, this can be this can be problematic in situations where the exposure variable is mis-specified i.e., the genetic instrument is biologically related to an intermediate or outcome, but has been identified as being related to the exposure by virtue of the latter's correlation with the biologically related trait (4), termed correlated pleiotropy (44). For example, although a locus in *FTO* was initially identified in relation to type II diabetes, this was subsequently found to primarily influence BMI with secondary effects on type II diabetes (45), leading to difficulties in interpreting MR studies where *FTO* variation is used as instrumental variable for type 2 diabetes.

In contrast, horizontal pleiotropy (i.e., a genetic variant is associated with two traits which influence the outcome via independent biological pathways) violates the exclusion restriction criterion (Figure 2B). GWAS identify genetic instruments purely on statistical grounds. Even if instrumental variables used in MR studies intersect genes with plausible pathways to the exposure it's not possible to be sure whether they mediate the causal effect being evaluated. Therefore, potential horizontal pleiotropy as a result of unknown pathways needs to be excluded if MR studies are to reach robust conclusions about causality. One simple method to limit the impact of horizontal pleiotropy is leave-one-out as a sensitivity analysis to ensure that the causal effect is not mediated by an outlier effect of one specific locus (46).

Over the last few years, several methods have been developed that relax the strict requirement that genetic instruments exhibit no horizontal pleiotropy yet still produce consistent causal effect estimates (7). One such approach is MR-Egger regression (47), where given a set of genetic variants that proxy an exposure variable of interest, a regression is performed between estimates of the SNP-outcome association and SNP-exposure association (this can be performed in both one and two-sample MR analyses). Unfortunately, Egger regression is limited by very poor power. Weighted median and weighted mode approaches have since been developed to derive causal estimates based on the relationship between the strength of the association between the SNP and the outcome, and the strength of the association of the SNP with the exposure, which are more robust to violation of horizontal pleiotropy by a substantial proportion of instruments (48, 49). Several additional methods now exist which assume that only a certain proportion of the genetic instruments have a horizontal pleiotropic effect. These methods aim to reduce heterogeneity by removing SNPs that contribute to heterogeneity disproportionately, based on the standard errors of the Wald ratios. Such outlier removal strategies are applied in the MR-PRESSO (50), and generalized summary MR (GSMR) approaches (51). One of the issues in applying MR methods that are robust to pleiotropy is that in order to detect causal effects these require large sample sizes (49). Another issue concerns the number of SNPs used as IVs; significant numbers of SNPs are required to provide sufficient data points for meaningful analysis. However, an advantage is that these approaches often rely on different sets of assumptions, and if consistent, conclusions can be drawn regarding causality with reasonable confidence.

The recent review by Lawlor et al. provides a useful summary of the various methods and extensions of Mendelian Randomization (52). Some of these more advanced MR analysis methods have been applied in relatively recent studies examining causal inference in osteoporosis. For example, in their study examining causal relationships between blood lipids and eBMD, Cherny et al. found broadly similar inverse associations between LDL-C and eBMD as assessed by inverse-variance weighted (IVW), MR-Egger, weighted median and weighted mode estimates (53). Similarly, in our recent study, MR-Egger, IVW and weighted median estimates showed similar causal effects of BMD on sclerostin (54). That said, the statistical evidence against the null for the MR-Egger estimates was somewhat lower, in both these papers, reflecting the lower statistical power of this test (48, 49). However, these types of sensitivity analyses have been lacking in many of the MR analyses in osteoporosis, including those analyzing a range of metabolites for which genetic influences could well-exert pleiotropic effects via unknown pathways (38).

It may be hard to exclude pleiotropy where the genetic instruments comprise just one or two SNPs, however there are exceptions to this. For example, we recently performed an MR study to examine the causal relationship between sclerostin levels and eBMD, based on results of a sclerostin GWAS where we identified just two loci. However, we were able to establish a causal relationship between sclerostin and eBMD using co-localization analysis, which interrogates LD structure at a single locus, in this case the gene encoding B4GALNT3 (54).

Several analysis methods have been developed to explore causal pathways in those situations where genetic instruments are pleiotropically associated with multiple correlated phenotypes. For example, in studying the causal relationship between genetically determined BMD (reflected by eBMD) and OA, Funck-Brentano et al. observed a strong causal effect of BMI on knee and hip OA, suggesting that if any eBMD SNPs are shared with BMI, this may influence OA via pathways other than BMD, which will violate the 3^rd^ MR assumption (horizontal pleiotropy). The authors established that genetically determined BMD also has a causal effect on OA after excluding pleiotropic pathways involving BMI, by removing SNPs from their eBMD polygenic risk score that were related to BMI (55). Similarly, Cousminer et al. excluded SNPs for height and BMI in their MR analysis of the causal role of pubertal age on BMD (33).

An alternative method of accounting for pleiotropy where genetic variants are pleiotropically associated with multiple correlated phenotypes, is to perform multivariable MR. The latter aims to address this limitation by using instruments associated with multiple exposures to jointly estimate the separate causal effect of individual risk factors on the outcome (34, 56–58). For example, Kemp et al. used a one-sample multivariable method to show that BMI SNPs acted via both lean and fat mass to increase BMD (34). In an MR analysis of relationships between plasma lipids and BMD, we observed a strong inverse association between LDL cholesterol and forearm BMD in multivariate MR analyses adjusting for HDL cholesterol and triglycerides, which was not evident in univariate analyses involving only LDL cholesterol (59). This indicates that complex relationships may exist between the causal effects of different lipids and BMD, which MR analyses need to account for, and may help to explain the conflicting results from different MR analyses examining relationships between lipid levels and eBMD in the UK Biobank (53, 59, 60).

## Distinguishing Genetic Correlation From Causality

Key points:-

1. Traits which are correlated as a result of shared underlying biology are likely to have shared genetic influences, leading to a positive signal in MR studies
2. MR signals arising from genetic correlation between two traits are expected to be bidirectional; true causal effects generally produce a positive MR signal in one direction only (i.e., exposure to outcome as opposed to outcome to exposure)
3. In bidirectional MR, it may be helpful to use methods such as Steiger filtering to restrict SNPs to those which have strongest effects on the outcome as opposed to the exposure being tested
4. Though rarer, bidirectional causal effects may exist, exemplified by a positive causal effect of BMD on sclerostin levels, and a negative causal effect of sclerostin on BMD.

It's common for two related traits to share a proportion of their heritability, as quantified by genetic correlation, implying some form of shared underlying biology. Bidirectional MR can help distinguish causality from correlation by first testing the associations in one direction (i.e., “exposure” to “outcome”), and then performing these in the opposite direction (i.e., “outcome” to “exposure”), using SNPs found to be associated with each trait in different GWASs. In those instances where certain SNPs are common to GWASs for both the exposure and outcome, methods such as Steiger filtering are recommended to remove these SNPs to ensure they are used correctly as instruments for analyses in one direction only (61). Bidirectional MR assumes that the underlying causal association works in a single direction. Where there is evidence for “bidirectional causality,” this may simply reflect genetic correlation arising from a common genetic pathway affecting both the exposure and outcome. That said, bidirectional Mendelian randomization can identify causal effects that do work in both directions; for example, smoking reduces BMI and higher BMI increases smoking (62). In the case of bidirectional causality where evidence is stronger in one direction, although the main causal pathway may be in this direction, findings may reflect misspecification of the exposure variable as described above. Alternative strategies to MR, such as latent causal variable analysis, have been developed to distinguish correlation from causality (63).

Certain biomarkers and risk factors for osteoporosis may be unlikely to show strong genetic correlations with BMD, and to be influenced by common biological pathways, nevertheless it's still good practice to perform bidirectional MR. For example, in the case of factors such as smoking, which was found to be genetically related to lower heel BMD (25), in the absence of bidirectional MR, it's not possible to exclude reverse causality, which is not inconceivable given the casual effect of BMI (which is known to influence BMD) on smoking (62). In recent studies examining relationships between panels of blood metabolites and BMD, where the direction of the causal effect is unclear, whereas one study reported findings from bidirectional analysis (64), a further one did not (38). In addition, genetic correlation as a consequence of shared biological pathways could conceivably explain relationships between BMD and other disease phenotypes such as osteoarthritis (OA). For example, a previous study revealed significant genetic correlation between LS BMD and hip and/or knee OA, suggesting common genetic influences, exemplified by the *SMAD3* locus found to affect both OA risk and BMD (65). Shared genetic influences on BMD and OA could also explain recent findings that genetic instruments for eBMD are associated OA (55); whereas the authors interpreted this as indicative of a causal effect of eBMD on OA, bidirectional MR is required to prove such a causal pathway exists, as opposed to common biological mechanisms contributing equally to both traits.

One of the challenges in performing bidirectional MR between two variables which are highly correlated genetically is that the two traits are likely to share one or more SNPs in common. This is particularly an issue when using results derived from large GWAS studies that generate many signals. For example, in our recent study of relationships between eBMD and lipids, a bidirectional effect for eBMD on LDL-C was investigated using 404 eBMD associated SNPs as genetic instruments (12). Steiger filtering was used to identify SNPs that had stronger effects on the outcome (LDL-C) compared to the exposure (BMD). This analysis suggested that 394 of 404 SNPs exerted their primary effect on BMD as opposed to LDL-C levels. IVW MR, weighted median MR and MR-Egger regression results showed some evidence that eBMD might influence LDL-C, and the association remained unchanged after Steiger filtering to remove those SNPs that primary affected LDL-C levels (59).

As well as selectively removing SNPs to assist interpretation of bidirectional analyses, this approach may also be helpful in examining the role of specific biological pathways involved in mediating causal effects. For example, in the above MR analysis of the effects of plasma lipids on eBMD, we were able to confirm that the inhibitory effect of LDL cholesterol on eBMD which we observed was not solely mediated by SNPs intersecting the *HMGR* locus which is the target for statin therapy, since similar results were obtained when SNPs at this locus were removed from the polygenic risk score. Similarly, SNPs can be stratified into relevant/specific biological pathways and their association with outcomes of interest tested. For example, although not a formal MR analysis, Warrington et al. used genetic risk scores constructed from SNPs belonging to specific biological pathways, and showed that genetic risk scores comprising variants that belonged to the RANK-RANKL-OPG pathway, the mesenchymal stem cell differentiation functional pathway and the WNT signaling function pathway were associated with bone measures at age 13, but only mesenchymal stem cell differentiation and the WNT pathway SNPs showed associations with rate of change in BMD between 9 and 17 years (66).

**Figure 3:**
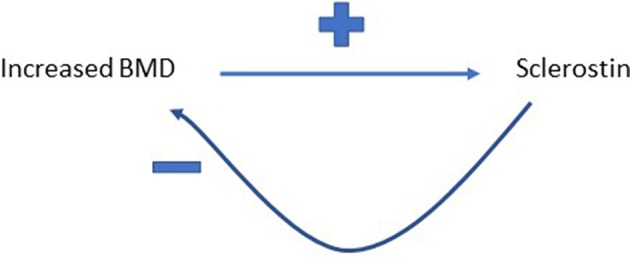
Proposed feedback pathway between BMD and sclerostin. Greater BMD is proposed to increase circulating levels of sclerostin, which then feeds back to inhibit bone formation, and hence limit further gains in BMD.

It's a reasonable assumption that correlated variables as a result of shared biology show equivalent “causal” effects on bidirectional MR, whereas for a true causal relationship, an effect is just observed in one direction. However, we recently observed a further pattern in our study exploring the relationship between circulating sclerostin levels on eBMD, namely bidirectional causal pathways in opposite directions (54). We found that higher levels of serum sclerostin were causally related to lower FN BMD, lower eBMD and higher fracture risk. In contrast, greater BMD was causally related to higher sclerostin levels, using BMD SNPs identified in the GEFOS BMD GWAS (54). This finding aligned with the observational relationship between BMD and sclerostin we reported in the same paper and may be a reflection of a previously unsuspected negative control feedback mechanism for BMD (see Figure 3). However, the exact mechanisms involved remain unclear and functional validation of such a pathway is still needed.

## Power Considerations

In contrast to conventional epidemiological studies where the exposure variable comprises the population variance of the trait of interest, in MR, genetic instruments only capture a small proportion of trait variance (not infrequently <1%). As a consequence, the strength of the relationship between an instrumental variable used for MR, and the outcome of interest, will only be of a small fraction of that seen for the measured exposure variable. Therefore, limited power is a common problem for MR analyses, and a frequent explanation of null findings, and needs to be an important consideration particularly when the findings fail to support other well-established lines of evidence. Limited power is even more problematic for some of the more recent extensions to MR, such as multivariable MR.

In any given MR study, the major factors governing the power are the sample size, the strength of the genetic instruments available, the strength of the underlying causal relationship being evaluated, and the type I error rate. Recent availability of very large datasets, such as the UK Biobank, have facilitated well powered MR studies, as have the increasing number of GWAS signals available for any given trait. Nonetheless, even where a large sample is available, MR may be uninformative where available instruments are lacking, or if there is a weak underlying relationship. These considerations are particularly important when null associations are obtained, where it is helpful to report power calculations to illustrate the strength of any underlying relationship which would have been detectable, and how this compares with that seen in observational studies (67).

A related issue is weak instrument bias. In one-sample MR, using weak instruments may bias the causal association toward the observational association between the exposure and outcome, whereas in two-sample MR, weak instruments may bias MR estimates toward the null (68). Therefore, it is important to avoid such bias by evaluating instrument strength at the outset of the study. For most human phenotypes, common genetic variants only explain a limited proportion of the variance of the phenotypes; combining small effects across these common variants into a score (known as the polygenic risk score) may increase instrument strength (69). Another relevant concept is the “No Measurement Error” (NOME) assumption of MR, which assumes the association between a given genetic instrument and the exposure is estimated without measurement error (70). This is particularly important when weak instruments are estimated from GWAS with small sample sizes. In an IVW setting, the mean F-statistic can be used to assess whether instruments violate the NOME assumption, a value below 10 implying a high likelihood of weak instrument bias (71). In an MR-Egger setting, the I2 statistic (between 0 and 1) can be used to quantify violation of the NOME assumption, a lower value indicating greater likelihood that this assumption has been violated (48).

In the majority of instances of null findings reported in recent MR studies of osteoporosis risk factors, genetic instruments have been identified based on genome-wide significant associations from large scale GWASs, and although instrument strength is not universally reported, weak instrument bias is less likely to be an issue under these circumstances (70). However, weak instrument bias may be an issue in those instances where instruments have been identified from relatively small GWAS studies. For example, in a two-sample MR study examining causal relationships between inflammation and BMD reporting null findings, three of the genetic instruments for IL-6 were derived from a population of 1,664 individuals, and had F-statistics ranging from 3 to 8, indicating high likelihood of weak instrument bias (29).

## Future Directions

It may be possible to extend MR to identify novel risk factors for osteoporosis using a hypothesis-free approach. For example, centralized databases such as MR-Base (46) and UK Biobank (http://www.nealelab.is/uk-biobank) have harmonized GWAS summary results for more than 20,000 complex human traits. Such resources make it feasible to conduct a phenome-wide MR for osteoporosis, aimed at identifying novel causal effects on BMD from screening a comprehensive range of complex traits. In many cases, mega biobanks such as UK Biobank, provide the richest source of GWAS-linked exposure or outcome data. Consequently, the issue of overlapping samples for generating genetic instruments and providing outcome data in a two-sample MR framework, potentially providing biased estimates (8), is becoming increasingly problematic. With the burgeoning opportunities for performing MR analyses, there also comes the need to ensure these are performed and reported comprehensively, with thorough exploration of issues such as pleiotropy, reverse causality and power, to ensure appropriate conclusions are drawn. STROBE-MR guidelines, intended to improve the quality of reporting of MR studies, have recently been produced (72).

MR was initially developed to examine the causal role of environmental exposures on the outcome of interest. This method has since been applied to a wide range of research areas, including drug target validation and prioritization, and the interpretation of multi-dimensional omics data. Large-scale GWASs of omics data, such as metabolites, DNA methylation, gene expression and protein expression provide a timely opportunity to identify the causal relationship of thousands of molecular phenotypes with osteoporosis in a MR framework. Automated tools such as summary-data-based MR (SMR), Generalized Summary-data-based MR (GSMR) and the two-sample MR R package make it possible to conduct such large-scale analyses effectively (46, 51, 73).

For omics studies of osteoporosis, one of the issues that needs further consideration is tissue specificity. Most molecular phenotypes to date have been measured in whole blood, for which the sample size of expression QTLs and methylation QTLs studies exceeds 30,000 (74) (http://www.godmc.org.uk/) and protein QTLs studies exceed 6,000 (75, 76). In contrast, the QTLs measured in bone tissues are limited to several hundreds of individuals. Whether molecular phenotypes measured in blood can be used as a proxy for those measured in bone tissues remains unclear, particularly methylation which shows a high degree of tissue specificity, in line with emerging trends in tissue specific MR (77), implying an urgent need for osteoblasts, osteoclasts and osteocytes and other skeletal cell types to be sufficiently well- represented in omics resources.

## Conclusions

MR is being increasingly applied to examine causal inference in osteoporosis, reflecting the increasing availability of large datasets such as the UK Biobank, and multiple GWASs for potential risk factors. To date, the most important findings have been around the lack of causal role of traditional risk factors such as vitamin D in determining variation within the normal range of BMD/fracture risk. High-dimensional omics studies, based on GWASs of metabolites, gene expression and DNA methylation, offer exciting opportunities for future discovery, with the emergence of the first MR studies of metabolites in osteoporosis. However, an important caveat is that MR studies can be complicated by a number of issues including horizontal pleiotropy, reverse causality, and lack of power. Several extended MR methods have been developed to explore these aspects, and while not always applied consistently, r STROBE-MR guidelines have recently been produced, intended to support the quality with which MR studies are reported.

## Author Contributions

JZ and MF helped to write the manuscript. JK, DE, and GS reviewed the manuscript and provided critical comments. JT conceived and helped to write the manuscript.

### Conflict of Interest

The authors declare that the research was conducted in the absence of any commercial or financial relationships that could be construed as a potential conflict of interest.
